# A Case Series of Concomitant Cardiac Electrical Disease among Takotsubo Syndrome Patients and Literature Review

**DOI:** 10.3390/jcdd9030079

**Published:** 2022-03-09

**Authors:** Ibrahim El-Battrawy, Julia W. Erath, Mate Vamos, Assem Aweimer, Andreas Mügge, Siegfried Lang, Uzair Ansari, Thorsten Gietzen, Ibrahim Akin

**Affiliations:** 1 First Department of Medicine, Faculty of Medicine, University Medical Centre Mannheim (UMM), University of Heidelberg, 68167 Mannheim, Germany; siegfried.lang@umm.de (S.L.); uzair.ansari@umm.de (U.A.); thorstengietzen@icloud.com (T.G.); ibrahim.akin@umm.de (I.A.); 2 DZHK (German Center for Cardiovascular Research), Partner Site, 69120 Heidelberg, Germany; 3 Bergmannsheil Bochum, Medical Clinic II, Department of Cardiology and Angiology, Ruhr University, 44801 Bochum, Germany; assem.aweimer@bergmannsheil.de (A.A.); andreas.muegge@bergmannsheil.de (A.M.); 4 Department of Cardiology, Division of Clinical Electrophysiology, University Hospital Frankfurt, Goethe University, 60323 Frankfurt, Germany; julia.erath@gmx.net (J.W.E.); mate.vamos@uni-frankfurt.de (M.V.)

**Keywords:** Takotsubo Syndrome, acquired LQT syndrome, catecholamine excess, mortality, estrogen, beta-blocker

## Abstract

The pathophysiology of Takotsubo Syndrome (TTS) is not completely understood and the trigger of sudden cardiac death (SCD) in TTS is not clear either. We therefore sought to find an association between TTS and primary electrical diseases. A total of 148 TTS patients were analyzed between 2003 and 2017 in a bi-centric manner. Additionally, a literature review was performed. The patients were included in an ongoing retrospective cohort database. The coexistence of TTS and primary electrical diseases was confirmed in five cases as the following: catecholaminergic polymorphic ventricular tachycardia (CPVT, 18-year-old female) (*n* = 1), LQTS 1 (72-year-old female and 65-year-old female) (*n* = 2), LQTS 2 (17-year-old female) (*n* = 1), and LQTS in the absence of mutations (22-year-old female). Four patients suffered from malignant tachyarrhythmia and recurrent syncope after TTS. Except for the CPVT patient and one LQTS 1 patient, all other cases underwent subcutaneous ICD implantation. An event recorder of the CPVT patient after starting beta-blocker did not detect arrhythmias. The diagnosis of primary electrical disease was in 80% of cases unmasked on a TTS event. This diagnosis triggered a family clinical and genetic screening confirming the diagnosis of primary electrical disease. A subsequent literature review identified five cases as the following: a congenital atrioventricular block (*n* = 1), a Jervell and Lange-Nielsen Syndrome (*n* = 1), and a family LQTS in the absence of a mutation (*n* = 2), LQTS 2 (*n* = 1). A primary electrical disease should be suspected in young and old TTS patients with a family history of sudden cardiac death. In suspected cases, e.g., ongoing QT interval prolongation, despite recovery of left ventricular ejection fraction a family screening is recommended.

## 1. Introduction

Takotsubo Syndrome (TTS) is a reversible acute heart failure caused by a cardiac dysfunction and wall motion abnormalities mimicking acute coronary syndrome. Recently published data sorted TTS into four forms: the apical, the midventricular, and the rarely described basal and focal forms [1,2,3]. Different electrocardiogram changes, including ST-segment elevation, inverted T-waves, long QT syndrome (LQTS), and Brugada syndrome (BrS)-like ECG changes, have been described [4,5]. Recently, data mimicking the TTS model in vitro using cardiomyocytes from induced pluripotent stem cells presented that estradiol may attenuate susceptibility to developing LQTS [6]. The predominance of TTS in postmenopausal females may enhance the speculation that low estrogen levels may be linked to the pathomechanism of TTS. LQTS is regularly described as an acquired form in TTS in the acute as well as subacute stages. Recently published data presented the mortality of TTS as being comparable to patients suffering from acute coronary syndrome. This high mortality is explained by TTS-associated complications such as fatal arrhythmias in up to 14% of TTS cases, mostly triggered by a QT prolongation [7,8,9,10]. Acquired LQT syndrome can cause life-threatening ventricular arrhythmia, such as Torsade de Pointes, with higher risk for sudden cardiac death (SCD). However, understanding the arrhythmic substrate and triggering factors of TTS remains lacking. It is not yet clear if only the reduced left ventricular ejection fractions explain the risk of malignant arrhythmias or other pathomechanisms that are linked to this issue. Additionally, data have shown that TTS patients may suffer from malignant arrhythmias and/or SCD several months after the TTS event and/or after recovery of left ventricular ejection fraction [11,12]. In addition, case reports reported Brugada syndrome ECG changes in TTS patients [5].

The purpose of this retrospective study was to detect a possible association between congenital channelopathy in the presence of TTS in a bi-centric study as well as a literature review of published cases.

## 2. Methods

A cohort of 148 patients presenting with TTS was reviewed and included in an ongoing retrospective cohort database from 2003 to 2017. They were part of the daily patient collective of the University Medical Centre Mannheim and the University Medical Centre Frankfurt. Additionally, a subsequent literature search using PubMed and the MeSH terms “Takotsubo” and “Congenital” identified further cases.

Patients were presenting with symptoms of an acute coronary syndrome as chest pain or dyspnea, including electrocardiographic changes and the release of troponin and other myocardial enzymes, in the absence of significant obstructive coronary artery disease and fulfilled the Mayo Clinic criteria for TTS [13].

All patients underwent coronary angiography to exclude a significant obstructive coronary artery disease. Demographics; symptoms on presentation; electrocardiographic and transthoracic echocardiography findings; and cardiac enzymes including creatine, troponin I, and creatine phosphokinase were recorded during the hospital admission. In selected cases, a cardiac magnetic resonance tomography (CMRI) was performed. Myocarditis and pheochromocytoma were excluded before reaching a diagnosis of TTS, as described previously [14].

The angiograms, echocardiograms, and ECGs were reviewed by two experienced independent cardiologists to evaluate the diagnosis of TTS. The study protocol was approved by the Ethics Committee of University Medical Centre Mannheim (approval number 2015-841R-MA). All methods were performed in accordance with the relevant guidelines and regulations.

In-hospital events, arrhythmias, cardiac rupture, thromboembolic events, pulmonary congestion with use of non-invasive positive-pressure ventilation, intubation, use of a temporary pacemaker, use of inotropic agents, and in-hospital death were assessed based on chart review. If medical records, treating physicians, or relatives were unable to provide further information concerning the circumstances of death, it was defined as death due to unknown cause.

## 3. Statistical Analysis

Data are expressed as mean values ± standard deviation for continuous variables with a normal distribution, median (interquartile range) for continuous variables with a non-normal distribution, and percentages for categorical variables.

Statistical analysis was performed using SPSS 23.0 in all analyses; *p*-values < 0.05 (two-tailed) were considered statistically significant.

## 4. Results

Among 148 TTS cases, five patients suffered from different channelopathies.

### 4.1. TS and Catecholaminergic Polymorphic Ventricular Tachycardia

**Figure 1:**
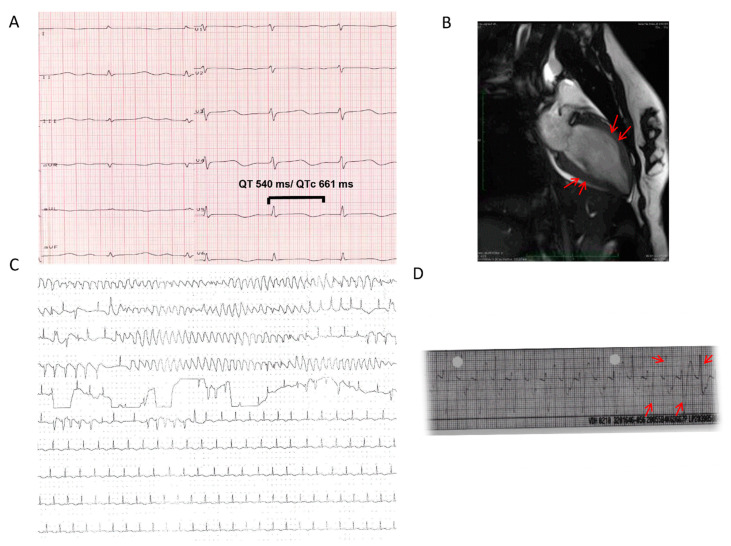
(**A**) ECG at TTS event of 72-year-old female patient presented prolonged QTc interval at TTS event. Due to intolerance of beta-blockers and ongoing prolonged QTc interval in regular follow-ups, an s-ICD has been implanted. A genetic screening confirmed a mutation in the KCNQ1 gene. (**B**) Cardiac magnetic resonance of 18-year-old female patient with a bidirectional tachycardia within the operative rhinoplasty and a family history of sudden cardiac death: aunt at 36 years old. It shows a midventricular ballooning consistent with TTS. (**C**) Holter ECG in the intensive care unit of a female patient with a previously diagnosed LQTS detected a recurrent ventricular tachycardia. Cardiac magnetic resonance confirmed a coexistence of apical ballooning. (**D**) ECG presents a bidirectional tachycardia that has terminated after 20 min.

An 18-year-old female patient was admitted for rhinoplasty. Her family history was associated with unexplained SCD of her aunt at 36 years of age, and the patient had 10 episodes of unexplained syncope during physical stress. General anesthesia was performed using propofol and remifentanil. However, after that, the patient developed a systolic blood pressure <70 mmHg associated with bidirectional tachycardia (Figure 1D) between 150 and 170 bpm, which was terminated spontaneously after 20 min and converted to sinus tachycardia. Due to elevated cardiac enzymes, coronary angiography, echocardiography, and cardiac magnetic resonance imaging were performed (Figure 1B). This confirmed midventricular TTS with an ejection fraction (EF) of 40%. Recovery of the EF was documented within 7 days. The patient was started on treatment with a beta-blocker, and thereafter, no arrhythmias have been documented. A detailed family screening confirmed a case of sudden cardiac death: her aunt at the age of 36 years. However, no CPVT-related genes were documented using next-generation sequencing.

### 4.2. TTS and LQTS 1

#### 4.2.1. Family One

**Figure 2:**
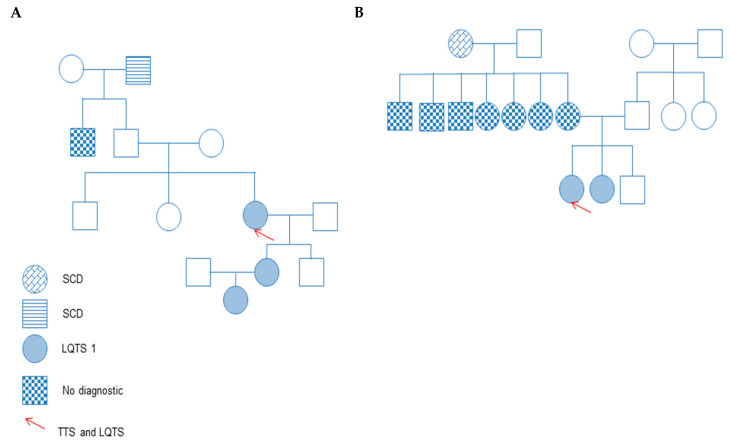
(**A**,**B**) Family pedigrees of two TTS patients affected by LQTS 1 at random after a detailed screening due persistence of ECG changes despite recovery of TTS.

A 72-year-old woman was admitted for a nasal septoplasty. One day after the surgery, the patient complained about chest pain. Cardiac enzyme levels were elevated. A coronary angiography was performed, and an acute coronary syndrome was invasively ruled out. However, subsequent ventriculography and transthoracic echocardiography showed an apical ballooning and LQTS (QTc 661 ms) in her 12-lead-ECG (Figure 1A), consistent with TTS and highly impaired LVEF 33%. A regular check-up showed a recovery of her EF, but QTc prolongation persisted electrocardiographically. The patient was treated with a beta-blocker. Genetic screening was performed to exclude a coincident primary electrical disease. However, the result proved a novel LQTS type 1 heterozygous mutation in KCNQ1 (3bp-Deletion c. 1084_1086delAAG). Five weeks after hospital discharge, the QTc interval was normalized. Family clinical and genetic screening confirmed a familial LQTS I. Due to recurrent syncope of the patient and intolerance of the beta-blocker, a subcutaneous ICD was recommended and implanted. A family screening due to one or two syncope episodes confirmed a LQTS 1 in more than member (Figure 2A). They were started with a treatment of propranolol.

#### 4.2.2. Family Two

A 65 year-old woman was admitted to the hospital due to dyspnea. She has a history of chronic obstructive pulmonary disease. Due to elevated cardiac enzymes, a coronary angiography was performed and excluded coronary artery disease. However, laevo-cardiography and echocardiography showed a midventricular TTS form with QTc interval prolongation (660 ms). Metoprolol was started. However, due to persistent QTc prolongation after recovery of LVEF, a family screening was started to exclude congenital long QT syndrome. Sudden cardiac death was documented (Figure 2B). A further family screening confirmed a congenital long QT syndrome with a mutation in KCNQ1 (NM_000218.2:c.775C>T, p.(Arg259Cys), Exon 5) consistent with LQTS 1. Her sister has had three syncope episodes.

### 4.3. TTS and LQTS 2

A 17-year-old girl attended a concert and was successfully resuscitated in the field from cardiac arrest due to ventricular fibrillation, leading to emergency hospital admission. The patient suffered from recurrent syncope. Her mother was diagnosed with an LQTS 2 several years ago. In the acute setting, coronary artery disease was ruled out invasively. An additionally performed ventriculography demonstrated an apical TTS form and impaired LVEF of 30%. The ECG showed significant QT prolongation (QTc = 500 ms). The patient was treated by a beta-blocker and was fitted with a wearable cardioverter defibrillator (WCD) after the intensive-care unit (ICU) stays. Three months later, left ventricular function was normalized. A subcutaneous ICD was implanted for secondary prevention.

### 4.4. TTS and LQTS

A 22-year-old patient was admitted to the hospital due to out-of-hospital cardiac arrest (OHCA) because of ventricular fibrillation (VF). An LQTS was diagnosed four years ago. An acutely performed coronary angiography excluded a coronary artery disease. However, an echocardiography documented a highly reduced EF with apical and midventricular wall motion abnormality. In the ICU, recurrent ventricular tachyarrhythmias were documented (Figure 1C). A cardiac magnetic resonance tomography confirmed an TTS of apical form. The EF was recovered within 7 days, and a subcutaneous ICD was implanted for secondary prevention.

### 4.5. Literature Research

Our literature research revealed five further TTS cases with a coincident primary electrical disease (Table 1). Mahida and colleagues [11] presented a case of a 55-year-old female. Despite EF recovery due to medical therapy, the patient presented ongoing QTc interval prolongation. A genetic screening of KCNQ1, KCNH2, SCN5A, KCNE1, and KCNE2 failed to identify any known mutation. She commenced with a beta-blocker.

Another 37-year-old [12] patient was admitted with TTS and prolonged QTc interval. TDP was recorded during intensive care unit stay. Follow-up over 4 years demonstrated persistently prolonged LQTS. Genetic screening confirmed a novel mutation in the KCNH2 gene (nc.343–363dup corresponding to p.V115-A121dup) consistent with LQTS 2. The patient was started with a beta-blocker.

Additionally, two cases have been reported with a coincidence of channelopathy and TTS.

A 55-year-old female patient was diagnosed with TTS and LQTS. The patient was numb, and a medical history analysis presented more cases of family deafness and LQTS consistent with Jervell and Lange-Nielsen Syndrome [13]. A 42-year-old female patient with a congenital complete atrioventricular block was admitted to the hospital with chest pain. Coronary angiography and echocardiography confirmed a TTS of apical form.

Wedekind and colleagues reported a case of a fatal combination of TTS, hypertrophic cardiomyopathy, and congenital LQTS. An 81-year-old female was diagnosed with TTS triggered by an emotional stress event after receiving the message of SCD of her 54-year-old son. She developed recurrent Torsade de Pointes and ventricular tachycardia. Because of the pronounced QT interval prolongation in this patient, ECG recordings during previous hospital stays revealed an LQTS. In the family screening, the living daughter was clearly diagnosed with LQTS (QTc 520 ms).

## 5. Discussion

We performed a retrospective clinical investigation in 148 TTS patients and a subsequent literature review and showed that (i) the coincidence of TTS and primary electrical diseases is not uncommon; (ii) the pathophysiology of LQTS in TTS might be multifactorial; and (iii) TTS with a concomitant primary electrical disease might increase the risk of ventricular arrhythmias.

In unclear cases of myocardial infarction with non-obstructed coronary arteries (MINOCA), TTS diagnosis can be assessed by CMRI. Data have shown that patients with MINOCA are at high risk of major adverse cardiac events (MACE) regardless of the underlying cause of MINOCA. However, patients with a diagnosis of acute myocardial infarction according to CMRI are at the highest risk of MACE [15].

In cases with persistent LQTS in TTS despite normalization of LVEF, a congenital LQTS should be evaluated. Here, we describe a case of two older patients with persistent LQTS despite the recovery of EF. In addition, two young patients with a family history of LQTS suffered from TTS. Genetic and family screening confirmed a family congenital channelopathy. The literature review confirmed our observation. Further cases of an old TTS patient and two young patients have unmasked the diagnosis of family LQTS.

LQTS is a known dominator of tachyarrhythmias. The presence of QT-prolonging drugs, electrolyte imbalances, and bradycardia provoke acquired LQTS [16,17,18]. However, the underlying mechanism of LQTS in TTS remains not completely understood.

Patients with TTS release intense catecholamine levels, which lead to myocardial stunning, coronary vasospasm, and oxidative stress. The catecholamine-mediated myocardial toxicity can be seen as repolarization abnormalities in standard 12-lead ECG, including T-wave inversions and QT prolongation [19]. Those changes have also been reported for other hyperadrenergic states, such as pheochromocytoma. However, changes in repolarization patterns due to higher catecholamine levels seem to be transient. We recently presented action potential prolongation using cardiomyocytes from human-induced pluripotent stem cells. Our data showed that estradiol protects catecholamine induced action potential prolongation duration [5].

In the current study, we focus on a second cause of ECG changes in TTS. Since some cases are triggered by primary electrical disease, it is not excluded that a significant part of TTS patients has some LQTS-associated genes. Primary channelopathies and TTS might delay cardiac repolarization. TTS was shown to be associated with altered intracellular Ca^++^-handling [20]. It can be speculated that prolonged action potentials within LQTS or high intercellular calcium concentration as common in CPVT might provoke intracellular calcium overload, which in turn might also facilitate the occurrence of TTS. The current data might explain a second part of the puzzle of repolarization abnormalities in TTS.

A strong correlation between myocardial edema and the extent of repolarization abnormalities that are often associated with a long QT interval in patients with TTS has been reported in TTS cohorts [20,21]. Furthermore, the possibility that TTS is secondarily developed due to a heart rhythm disorder is not excluded.

Life-threatening arrhythmias including atrioventricular-block, ventricular tachycardia, and ventricular fibrillation have been reported in up to 14% of TTS cases, mainly in the setting of QTc interval prolongation [8,10]. TTS is predominantly found in women, whereas life-threatening arrhythmias are more common in males [22]. Several data sets of TTS showed that males have more co-morbidities than females and in-hospital TTS-related complications are more common in males than females, consistent with a higher mortality rate [23]. Men more frequently developed acute kidney injury, ventricular arrhythmias, cardiac arrest, and respiratory failure. However, a primary channelopathy should be excluded, especially in young TTS patients and old patients with a family history of primary electrical disease and ongoing ECG changes.

The medical treatment of TTS remains challenging. Data have shown that the use of beta-blockers might not be helpful in TTS patients [4]. However, beta-blockers might be of beneficial effect in TTS patients who are suffering from primary electrical disease. Recently published review articles presented an in-depth evaluation of interaction between catecholamine excess and β-adrenergic receptor signaling, G-protein coupled receptor kinases, calcium hemostasis, myofilament physiology, cardiomyocyte gene expression, cellular electrophysiology, inflammation, metabolism, central and peripheral nervous system, autonomic nervous system, hormone factors, the recently used microRNA vaccines, and energetics through different underlying pathways [24,25,26]. Of note, two categories of TTS have been described, primary TTS cases and secondary TTS cases. Primary cases are admitted to the hospital due to symptoms related to heart disease. On the other hand, secondary TTS cases are admitted to the hospital due to other disease and these patients develop TTS within the hospital stay [27]. Data have shown that both categories are associated with different outcomes. A recently published pilot study did not identify a change of expression levels in beta-receptors among TTS patients [28].

A recently published literature review showed presence of TTS among patients who are suffering from COVID-19 with only a predominance of females and a high rate of critical illness in these patients [29]. Several case reports showed the admission of patients with repolarization abnormalities in ECG and TTS patterns after receiving COVID-19 vaccination [30,31]. In patients with COVID-19, another cardiac disease could affect the ventricle, e.g., myocarditis. No guidelines are available yet on diagnosis and treatment of these cases [32]. Of note, data have shown that a declining left ventricular function may be provoked by critical illness and is linked to an increased risk of death [33]. The prognostic importance of LV dysfunction in critical illness might be underestimated.

## 6. Study Limitation

There are some limitations of this study that need to be mentioned. First, we report a very small number of TTS cases with co-incidence of primary electrical diseases. The described institutional database consists of 148 patients, and most data were obtained by a retrospective bi-center observation with confined event rates. Only TTS patients with suspected congenital channelopathy have received genetic and family screenings. Therefore, it is not excluded that other cases of inherited channelopathies (e.g., LQTS) are underestimated in TTS patients due to historical data about acquired LQTS in TTS patients.

## 7. Conclusions

The results of this study may show that TTS could be associated with repolarization abnormalities including QT prolongation. However, they may explain that LQTS is not an acquired syndrome in TTS and could be a primary trigger of TTS. Further studies are needed to determine its pathophysiological significance and to develop multivariate risk stratification for life-threatening arrhythmias in TTS. In the case of ongoing QTc prolongation months after the TTS event, malignant arrhythmia, and/or history of sudden cardiac death in family, screening for QTc prolonging genes should be completed.

## Figures and Tables

**Table 1:** Illustration of all TTS cases showing a presence of primary channelopathy in our cohort and in published cases.

Coincidence of TTS and Primary Electrical Disease					
Sex, Age	Primary Electrical Disease	TTS Form	Diagnosis of Primary Electrical Disease	Arrhythmia at TTS and Treatment	Affected Gene
Female, 18	CPVT	midventricular	at TTS event	VT; Beta-blocker	none
Female, 72	LQTS1	apical	at TTS event	Beta-blocker and s-ICD	KCNQ1
Female, 17	LQTS2	apical	at TTS event	VF; Beta-blocker, s-ICD	not done
Female, 22	LQTS	apical	prior TTS event	VF; Beta-blocker, s-ICD	none
Female, 65	LQTS1	midventricular	at TTS event	Beta-blocker	KCNQ1
**Case reports of literature review**					
Female, 55	LQTS	apical	at TTS event	TDP; Beta-blocker	none
Female, 55	Jervell- and Lange-Nielsen-Syndrome	apical	at TTS event	None, none	not documented
Female, 42	Atrioventricular block III	apical	prior TTS event	TDP, ICD	not documented
Female, 37	LQTS2	apical	at TTS event	TDP, beta-blocker	KCNH2
Female, 81	LQTS	apical	at TTS event	TDP and VT, ICD	not documented

CPVT: catecholaminergic polymorphic ventricular tachycardia; LQTS: Long QT syndrome; TDP: Torsade de Pointes; s-ICD: subcutaneous ICD; VF: ventricular fibrillation.

## Data Availability

Are available by request.
